# Multiple Sclerosis Misdiagnosis: A Persistent Problem to Solve

**DOI:** 10.3389/fneur.2019.00466

**Published:** 2019-05-07

**Authors:** María I. Gaitán, Jorge Correale

**Affiliations:** Neurology Department, Fundación para la Lucha contra las Enfermedades Neurológicas de la Infancia (FLENI), Buenos Aires, Argentina

**Keywords:** multiple sclerosis, misdiagnosis, McDonald criteria, diagnostic error, multiple sclerosis mimics

Despite significant refinement in multiple sclerosis (MS) diagnosis in recent decades, no specific disease biomarker exists, as a result of which, confirming the diagnosis is not always a straightforward process. MS has heterogeneous clinical and imaging manifestations, which not only differ between patients, but also vary in individual patients over time. Disease signs and symptoms, presence of oligoclonal bands (OCB) and MRI findings have limited specificity, and misdiagnosis remains a problem with significant clinical and psychosocial implications for both patients as well as health care providers. Although the problem of MS misdiagnosis is known, true incidence and prevalence is not. Some data is available from case reports, and recent publications from specialized MS centers reported around 30% of cases originally referred for MS were finally diagnosed with other diseases (1). One study conducted in four academic MS centers revealed over 50% of patients carried a misdiagnosis for at least 3 years, 70% had received disease-modifying therapy (DMTs), and 31% experienced unnecessary morbidity as a direct result (2). A wide range of conditions can be mistaken for MS, including: migraine, cerebral small vessel disease, fibromyalgia, functional neurological disorders, and neuromyelitis optica spectrum disorders, along with uncommon inflammatory, infectious and metabolic conditions (1, 3). As early initiation of DMTs improves short- and long-term clinical outcome, pressure to establish timely diagnosis may also increase risk of misdiagnosis. Interestingly, revision of the McDonald criteria in 2017 emphasizes the problem of misdiagnosis risk in clinical practice (4). The McDonald criteria, first introduced for MS diagnosis in 2001, with revisions made in 2005, 2010, and 2017, incorporated MRI to fulfilled the criteria of dissemination in time (DIT) and dissemination in space (DIS), increasing early diagnosis sensitivity. However, McDonald criteria were not developed to distinguish MS from other conditions, but to identify MS or high likelihood of disease in patients with a typical clinical isolated syndrome (CIS) (i.e., optic neuritis, transverse myelitis, brainstem syndrome) (4).

In this sense, wrong application of the clinical criteria may: (1) include misinterpretation of a clinical symptom that is not caused by inflammatory demyelination as a demyelinating event, (2) acceptance of a historical episode of neurologic dysfunction, in the absence of contemporaneous or current objective evidence (neurologic examination, evoked potentials, or MRI) providing corroboration, and (3) overreliance on MRI abnormalities in patients with not specific symptoms (2).

Initial reports of MS misdiagnosis began at the end of the 1980s, interestingly, if MRI had been available, diagnostic error would have been prevented in most cases (5). However, when MRI became widely used, overestimations of radiological findings started to be reported (6). Despite introduction of radiological criteria for DIT and space DIS for both MS and for CIS (7), the problem has remained unsolved. A previous study showed that patients which were referred to MS centers for exclusive imaging findings suggestive of MS, they mostly did not have MS; most brain MRI T2 lesions were due to: migraine, age-related diseases, or hypertension (8). In more recent work, MRI contributors to MS misdiagnosis included: (1) overreliance on imaging abnormalities corresponding to DIS in patients with “non-specific neurologic symptoms,” (2) incorrect interpretation of a subcortical lesion as periventricular or juxtacortical to meet DIS criteria, and (3) misinterpretation of DIT due to variations in MRI slice orientation (2).

Many factors can help perpetuate misdiagnosis. For example: not reconsidering an established diagnosis even in the presence of certain atypical clinical or para-clinical features, so-called “red flags,” or incomplete evidence of MS. In this regard, many MS specialists after encountering atypical MS syndromes on several occasions, may consider them as part of the spectrum of disease; when patients are referred to a new clinician for non-medical reasons, the physician usually accept their pre-established diagnosis (9); also, contrary to what one may believe, not all patients are relieved to discover they do not have MS, and may experience resistance to a new diagnosis (10); last, neurologists not willing to admit medical errors or experiencing diagnostic uncertainty may also be a contributing factor.

MS misdiagnosis may increase morbidity as a result of psychological damage, risk associated with DMTs and corticosteroids use, inadequate treatment (2, 3), worsening of underlying disease such as in neuromyelitis optica spectrum disorders (NMOSDs; (11), or delay in treatment of other potentially curable pathologies. Misdiagnosed patients are also sometimes included in clinical trials, which can confuse trial results and expose patients to inappropriate treatment. Other important point to consider is the economic impact of treating misdiagnosed patients, particularly in developing countries.

Work is needed to prevent misdiagnosis; neurologists must look out for clinical findings or diagnostic test results raising red flags. Three main factors could help avoid misdiagnosis. First a correct McDonald criteria application, looking for typical clinical syndromes, or historical events backed by objective corroborative evidence. Second, an appropriate application of radiological McDonald criteria: correct classification of lesion topography (juxtacortical, periventricular, infratentorial, spinal cord), juxtacortical lesiones are next to the cortex, and periventricular lesions are situated around ventricles. Use of the central vein sign in susceptibility-weighted images, may play a role in discriminating MS from its radiological mimics (12, 13). MS diagnosis should not be based solely on MRI findings. In difficult cases, a second opinion from a neuro-radiologist is recommended. Finally, in cases with inconclusive diagnosis, presentation of a syndrome other than a typical CIS, or insufficient clinical or MRI evidence to support diagnosis, CSF and serum OCB assay should be performed (4). Isoelectric focusing and immunoblotting or immunofixation for IgG, significantly increase diagnostic sensitivity (95–100%) and specificity (86–87%) (14) (Table 1). However, it is important to remember that OCBs are not specific to MS. Conversely, findings atypical for MS in CSF suggest other diagnoses (22). In negative cases, an alternative diagnosis should be considered. Cervical MRI, in search of asymptomatic spinal cord lesions, should be run in these patients (23).

**Table 1:** Recommendations to prevent MS misdiagnosis.

**When Applying 2017 McDonald criteria:**			
The McDonald criteria were developed to diagnose MS or a high likelihood of the disease in subjects with a typical CIS after other diagnoses were excluded.			
Consider to postpone definitive diagnosis of MS when a categorical typical CIS is not present. Further clinical and radiological follow-up is recommended before a definitive diagnosis.			
Do not consider a previous event in the absence of current or contemporaneous objective evidence providing corroboration.			
A neurologist with experience in MS should integrate the clinical case with complementary studies in order to make a solid diagnosis.			
When clinical and brain MRI evidences are not enough for MS diagnosis, in patients with a progressive course at onset, in children, older individuals, and/or non-white populations, additional workout is needed including spinal cord MRI or CSF examination.			
**When atypical clinical syndromes and abnormal MRI findings:**			
Consider alternative diagnosis, common causes of misdiagnosis: migraine, fibromyalgia, conversion or psychogenic disorders, MRI changes due to vascular disease, NMOSDs			
**When discriminating MS from NMOSD and anti-MOG disease:** Consider the following aspects.			
	**MS**	**NMOSDs**	**Anti-MOG**
OCB	95–100% (14)	22% (15)	6–13% (16)
Anti-AQP4 (Ab)	Negative	70% (17)	Negative
Anti-MOG (Ab)	Negative	Negative	Positive (18)
EBV-Seroprevalence^*^	>99%(anti-EBNA-1, anti-EBNA-2, anti-VCA)	52% (anti-EA) (19)	9% (anti-VCA) (20)
MRZ (21)	78%	1–2%	1–2%

* *The laboratory techniques and the type of antibodies considered may account for the differences observed between the different groups. EA, Early antigen; EBNA, Epstein-Barr nuclear antigen; VCA, Viral capside antigen*.

Because patients with MS and NMOSDs have overlapping features care should be taken in this differential diagnosis, particularly in certain population such as African American, Asian, Latin American, and pediatric patients. Most patients with NMOSDs have detectable serum antibodies that target the water channel aquoporin-4 (NMO-IgG), which facilitates an early diagnostic distinction between patients who have NMOSDs and those who have MS (17, 24). However, around 30% of patients presenting with features of NMOSDs are AQP4-seronegative, some of them may have antibodies reactive with myelin oligodendrocyte glycoprotein (MOG) (18). Nevertheless, the diagnostic sensitivity and specificity of anti-MOG antibody have not been fully validated. Because treatments of MS and NMOSDs are different, and disease modifying treatments for MS can induce severe exacerbations serological testing for AQP4 and MOG should be done in patients with features suggestive of NMOSDs, particularly in populations at high risk (25) (Table 1).

Recently, the detection of a polyspecific intrathecal humoral immune response against measles, rubella and varicela zoster virus called MRZ reaction has been shown to be a highly specific marker of MS, but only moderately sensitive (21). Interestingly, MRZ reaction was absent in virtually all patients with NMOSDs and anti-MOG antibody associated CNS demyelination analyzed so far (21, 26). However, it must be taken into consideration that, the clinical reliability of MRZ reaction in a given population may be influenced by the natural prevalence and the local vaccination coverage against these three viruses (Table 1).

Given that Epstein-Barr virus (EBV) seroprevalence in patients with MS is practically universal (27), EBV seronegative persons with symptoms suggestive of CIS/MS are not likely to be frequently identified (28). It is conceivable that in those cases, EBV seronegativity could represent a clinically useful biomarker for a diagnosis other than CIS/MS (29, 30) (Table 1).

Diagnostic certainty must be established before starting long-term, expensive disease-modifying treatment. Reliable diagnosis of MS or of an alternative disorder requires application of clinical judgment, and correct interpretation of patient history, physical examination, imaging results, and laboratory findings, as well as clinician with MS expertise. Sometimes, a correct diagnosis may require periodic open-minded reassessments. Efforts should be made to strike a balance between timely MS diagnosis, and avoiding misdiagnosis. Reporting MS misdiagnosis may help prevent future diagnostic errors.

## Author Contributions

MG designed the article, wrote the manuscript, approved the version to be published, and agreed for all aspects of the work. JC, critically reviewed for intellectual content, approved the version to be published, and agreed for all aspects of the work.

### Conflict of Interest Statement

MG has received reimbursement for developing educational presentations from Merck-Serono Argentina, Genzyme Argentina and Novartis Argentina, and Biogen-Idec Argentina, and travel/accommodation stipends from Biogen-Idec Argentina, Teva-Tuteur Argentina, Merck-Serono Argentina, and Novartis Argentina. JC is a board member of Merck-Serono Argentina, Biogen-Idec LATAM, and Merck-Serono LATAM, and Genzyme global. JC is a board member of Merck-Serono Argentina, Novartis Argentina, Genzyme LATAM, Genzyme global, Biogen-Idec LATAM, and Merck-Serono LATAM. He is part of the Steering Committee for the clinical trials of Ofatumumab (Novartis Global). JC has received reimbursement for developing educational presentations for Merck-Serono Argentina, Merck-Serono LATAM, Biogen-Idec Argentina, Genzyme Argentina, Novartis Argentina, Novartis LATAM, Novartis Global, and TEVA Argentina as well as professional travel/accommodations stipends.
